# Impact of chronic graft-versus-host disease on quality of life and cognitive function of long-term transplant survivors after allogeneic hematopoietic stem cell transplantation with total body irradiation

**DOI:** 10.1186/s13014-022-02161-9

**Published:** 2022-11-29

**Authors:** Isabella Gruber, Oliver Koelbl, Wolfgang Herr, Ernst Holler, Matthias Edinger, Daniel Wolff

**Affiliations:** 1 Department of Radiation Oncology, University Hospital Regensburg, Regensburg, Germany; 2 Department of Internal Medicine III, University Hospital Regensburg, Regensburg, Germany; 3 Leibniz Institute for Immunotherapy, Regensburg, Germany

**Keywords:** Total body irradiation, Acute myeloid leukemia, Chronic graft-versus-host disease, Allogeneic hematopoietic cell transplantation, Quality of life, Cognitive function

## Abstract

**Background:**

Total body irradiation (TBI)-based-conditioning before allogeneic hematopoietic stem cell transplantation (allo-HSCT) is standard of care in patients with acute myeloid leukemia (AML) but can cause long-term morbidity. Data on the impact of chronic Graft-versus-host disease (cGvHD) on cognitive function (CF) and quality of life (QoL) of long-term transplant survivors are sparse.

**Methods:**

We analyzed patient-reported outcomes focusing on progression-free AML patients and 1st allo-HSCT applying a standardized TBI-technique with an average dose rate of 4 cGy/min to the total body and lung shielding in case of doses > 8 Gy. Instruments included the Functional Assessment of Cancer Therapy-Bone marrow transplant (FACT-BMT, version 4), the FACT-Cognition Function (FACT-Cog, version 3) and the Patient Health Questionaire-4 (PHQ-4). We put focus on the impact of cGvHD and compared the results to normative data derived from the general population.

**Results:**

Out of 41 eligible patients contacted, 32 (78.0%) patients with a medium follow-up of 154 months (Interquartile range 113, 191 months) participated in the study. Eleven patients (34.4%) had active cGvHD, 11 (34.4%) resolved cGvHD and 10 (31.3%) never had cGvHD. Patients with active cGvHD had poorer FACT-BMT, FACT-Cog and higher PHQ-4 scores compared to patients with resolved cGvHD or who never had cGvHD. Outcomes were similar in patients with resolved cGvHD and those who never had cGvHD. Patients with active cGvHD had similar FACT-Cog, but lower FACT-BMT in comparison to normative data. However, the overall patient sample had similar FACT-BMT and FACT-Cog in comparison to normative data.

**Conclusion:**

Our data indicate that CF of long-term survivors upon TBI-based allo-HSCT is not impaired, even in the presence of active cGvHD. However, active cGvHD has a negative impact on QoL.

*Trial registration*  The local Ethics Board of the University of Regensburg approved this study (Number 20-1810_1-101).

## Background

Allogeneic hematopoietic stem cell transplantation (allo-HSCT) is a curative treatment modality for selected patients with acute myeloid leukemia (AML). Despite increasing survival rates, allo-HSCT can be associated with long-term morbidity and mortality [1]. The influence of total body irradiation (TBI) as part of the conditioning regimen and chronic graft-versus-host disease (cGvHD) on cognitive function (CF) and quality of life (QoL) of very long-term survivors is still unclear. In this study, we analyzed patient-reported CF and QoL focusing on long-term transplant survivors after 1st allo-HSCT applying a standardized TBI-technique as conditioning regimen. Since cGvHD can have a significant impact on QoL and CF [2] we analysed its impact as additional relevant covariable.

## Patients and methods

### Data collection

We analyzed patient-reported QoL, CF, and symptoms of depression and anxiety in patients with primary or secondary AML who received their 1st allo-HSCT with TBI-based protocols at the Department of Hematology of the University Hospital Regensburg between 1999 and 2017. All patients had a follow-up time of at least 2 years and were relapse-free for at least 2 years. The 2 year follow up was chosen since the primary aim was long term outcome and reports have indicated a protracted recovery of neurocognitive function [3]. Donors included matched sibling donors (MSD), matched unrelated donors (MUD), mismatched unrelated donors (MMUD) and haploidentical/mismatched related donors (MMRD). Source of stem cells were peripheral blood, bone marrow or cord blood. Patients completed the Functional Assessment of Cancer Therapy-Bone marrow transplant (FACT-BMT, version 4), the FACT-Cognition Function (FACT-Cog, version 3), the Patient Health Questionaire-4 (PHQ-4) and a questionnaire about sociodemographic data. All patients visited the Department of Hematology of the University Hospital Regensburg for routine follow-up visits. Clinical data including cGvHD status were abstracted from the medical charts of the Departments of Hematology and Radiation Oncology of the University Hospital Regensburg. Transplantation variables included gender, diagnosis, patient age, Karnofsky performance score (KPS), hematopoietic cell transplantation-comorbidity index (HCT-CI), as described by Sorror et al. [4], 2017 European LeukemiaNet (ELN) genetic risk stratification, as described by Döhner et al. [5], disease status, stem cell source, intensity of conditioning regimen, chemotherapeutic regimen, recipient and donor characteristics (donor type, donor age, HLA-compatibility, gender match, cytomegalovirus serostatus), GvHD prophylaxis and the use of rabbit anti-thymocyte globulin (ATG). Data closing was April 2021. The local Ethics Board of the University of Regensburg approved this study (Number 20-1810_1-101).

### Treatment plan

The choice of conditioning regimen was based on the oncologists´ discretion and dependent on patient age, disease risk and comorbidities. All patients included in the analysis received TBI as part of a complex conditioning regimen. TBI was performed in a consistent manner with an average dose rate of 4 cGy/min to the total body and lung shielding in case of doses > 8 Gy. Over the years, four treatment protocols were used (8 Gy TBI/Cyclophosphamide/Fludarabine, FLAMSA-RIC/Cyclophosphamide/4Gy TBI, 12 Gy TBI/Cyclophosphamide and 8 Gy TBI/Fludarabine). From 2000 to 2013, two Siemens Primus linear accelerators (Siemens Medical Systems, Inc., Concord, CA) were used for TBI, and from 2013 to 2017 two linear accelerators of type Elekta Synergy ™ with an Agility ™ head (Elekta Ltd, Crawley, UK) were applied. We proved clinically good dose distributions and similar parameters with both linear accelerators [6]. All patients received 6 megavoltage (MV) photon beams. Patients were treated with a twice-daily fractionation and a minimum of 6 h between fractions. Patients were lying down on a couch at the floor level in supine and prone positions to extend the source-to-skin distance. A plate of Makrolon® polycarbonate of 1 cm thickness was placed on a stand above of the patient to neutralize the skin sparing by the buildup effect. The low diameter in the neck region was compensated by using a bolus of plastic modeling mass. Eight rotational arcs were used per patient position. The average time to deliver each fraction was 50–60 min per side (supine and prone). Additional fixed beams were used in cranial and caudal direction to compensate for the effects of inverse square variation with increasing distance. Two individual lung shields of MCP96 of calculated thickness were designed in case of doses > 8 Gy to reduce the total dose to the center of the lung to 3.5 Gy in supine and prone positions (total dose of 7 Gy). Radio-oncologists contoured two individual lung blocks for each patient on a CT scan with a 1–2 cm margin between the edge of the lung on the CT film and the edge of the block. Lung blocks were tailored to avoid shielding of the vertebrae. MV-imaging verified the shielding positions. Areas of the chest wall that were shielded by the blocks were supplemented once a day with electron beams to achieve the full dose to the thoracic walls. The electron fields delivered a supplemented dose of 5 Gy for 12 Gy regimens. In vivo dosimetry was used to verify the dose delivery on several points on the patient´s body, demonstrating the uniformity of the dose distribution [6].

### QoL measures and other data sources

The FACT-BMT (Version 4.0) is a self-report questionnaire. The FACT-BMT combines the 27-item FACT-G total score (score range 0–108), an assessment of physical well-being (PWB, score range 0–28), social/family well-being (SWB, score range 0–28), emotional well-being (EWB, score range 0–24) and functional well-being (FWB, score range 0–28) with a 10 item Bone Marrow Transplant subscale (BMTS, score range 0–40) to evaluate self-reported concerns after transplantation. Patients rate on five-point Likert scale the frequency with which each concern was recognized in the past 7 days. The FACT-BMT-Trial Outcome Index (FACT-BMT-TOI, score range 0–96) is the sum of PWB, FWB and the BMTS-score. The FACT-BMT total score (score range 0–148) is the sum of the BMTS score and of the FACT-G total score. Higher scores indicate better QoL. The FACT-Cog (Version 3.0) is a validated measurement to analyze self-reported cognitive complaints in cancer patients. It includes perceived cognitive impairments (FACT-CogPCI, score range 0–72), impact of perceived cognitive impairments on quality of life (FACT-CogQoL, score range 0–16), comments from others (FACT-CogOth, score range 0–16) and perceived cognitive abilities (FACT-CogPCA, score range 0–28). Patients rate on five-point Likert scale the frequency of each complaint in the past 7 days. Higher scores indicate better QoL. All data are analyzed and expressed as mean according to the FACIT recommendations. The Patient Health Questionnaire-4 (PHQ-4) analyzes symptoms of depression and anxiety over the last 2 weeks on a 4 point Likert-type scale. Patients indicate if they feel nervous, anxious or on edge (item 1), if they are not able to stop and control worrying (item 2), if they have little interest or pleasure in doing things (item 3) and if they feel down, depressed or hopeless (item 4). The anxiety subscale (GAD-2) is the sum of the items 1 und 2 and the depression subscale (PHQ-2) is the sum of the items 3 and 4. In summary, there are four categories of psychological distress (None = 0–2, mild = 3–5, moderate = 6–8 and severe = 9–12). Patients with a GAD2 or PHQ2 of ≥ 3 are categorized as present for anxiety or depression. The presence and absence of cGvHD was extracted from the database of the Department of Hematology. Acute GvHD and cGvHD were defined according to described standard criteria [7–9]. Acute GvHD is classified as clinically significant at grade II-IV aGvHD. Patients have clinically active cGvHD (Group 1: Currently active inflammatory manifestations of cGvHD independently of the use of immunosuppression), resolved cGvHD (Group 2: All signs of clinically activity of cGvHD have disappeared, past history of cGvHD, no use of immunosuppression) or never had signs of cGvHD (Group 3: Never having cGvHD). The overall rate of completion was 100% for the FACT-Cog and the PHQ-4 as well as 98.1% for the FACT-BMT (Missing answers referred to satisfaction with sexual functioning).

### Statistical analysis

Transplant-related characteristics were presented as absolute and relative frequencies for categorical variables and as median and interquartile range (IQR) for continuous variables. The Mann–Whitney U-test was used for comparisons of continuous variables and the chi-square test of independence for categorical variables. Spearman's rank correlation coefficients were calculated to analyze the association of the FACT-BMT, FACT-Cog and PHQ-4. One-way analyses of variance (ANOVA) were performed to explore the effect of cGvHD on QoL outcomes. Post hoc group comparisons were done using the Tukey-test. Median follow-up time was estimated by using the reverse Kaplan-Meier method. Normative data (unadjusted means and standard deviations) of a general U.S. adult population of Brucker et al. [10] were used for comparisons with the FACT-G and normative data (means and standard deviations) of a French healthy population of Lange et al. [11] for comparisons with the FACT-Cog. We excluded AML patients < 30 years of age (n = 4) for comparisons with the normative data of Lange et al. [11] because of missing reference values for patients < 30 years of age. Comparisons between the normative data and the patient population were made using the one-sample t-test. A minimum clinically important difference (MCID) in QoL scores was defined as half of standard deviation (0.5 SD), as reported by Norman et al. [12]. Missing data were treated according to the manual scoring guidelines. We used the cutpoints of van Dyk et al. [13] to discriminate cancer-related cognitive impairment (Perceived cognitive impairment-score, FACT-CogPCI < 54) from the healthy population (FACT-CogPCI ≥ 54). All p-values were two-sided and p-values < 0.05 were considered as significant. Statistical analysis was performed using SPSS 26.0 (SPSS Inc., Chicago, IL, USA) and graphics were performed with Excel (2013, Microsoft Office).

## Results

### Patient and transplantation characteristics

95 patients were identified who received TBI-based-conditioning before 1st allo-HSCT between 1999 and 2017. 54 patients (56.8%) died after allo-HSCT and were not available for the evaluation of the patient-reported outcomes. The causes of death were relapse (n = 37, 68.5%), GvHD (n = 8, 14.8%), infections (n = 6, 11.1%) and other causes (n = 3, 5.6%). The median survival time of the overall population sample (n = 95) was 152 months (IQR 188, 120 months). All 41 long-term transplant survivors were contacted via post between December 2021 and April 2022. 32 patients (78.0%) provided written informed consent and completed the questionnaires. Nine non-responders (22.0%) were excluded from the analysis after two unsuccessful contact attempts (response rate: 78.0%). At the time of the evaluation, all participants were in complete remission of their initially diagnosed AML. Three patients relapsed after 1st allo-HSCT but were successfully treated with azacitidine (n = 1) and donor lymphocyte infusions (DLI; n = 2) and remained in remission with a follow-up of at least 24 months after successful completion of treatment (min 24, max 154). Table 1 shows the baseline transplantation characteristics of the participants (n = 32). The median follow-up time of all participants was 153 months (IQR 113, 191 months).

**Table 1:** Baseline transplantation characteristics

	All (n = 32)	Group 1: Never cGvHD (n = 10)	Group 2: Resolved cGvHD (n = 11)	Group 3: Active cGvHD (n = 11)	p-value
*Patient age at the time of transplantation, years*					
Median (IQR)	39 (26, 47)	42 (34, 49)	28 (21, 49)	40 (31, 48)	.199
*Patient age at the time of survey, years*					
Median (IQR)	53 (37, 62)	59 (45, 63)	41 (29, 60)	54 (36, 62)	.264
*Follow-up time, months*					
Median (IQR)	153 (113, 191)	174 (103, 231)	152 (113, 199)	151 (120, 182)	.623
*Gender, n (%)*					
Women	13 (41.0%)	3 (30.0%)	5 (45.5%)	5 (45.5%)	.712
Men	19 (59.0%)	7 (70.0%)	6 (54.5%)	6 (54.5%)	
*Diagnosis, n (%)*					
Primary AML	22 (68.8%)	7 (70.0%)	7 (63.6%)	8 (72.7%)	.895
Secondary AML	10 (31.3%)	3 (30.0%)	4 (36.4%)	3 (27.3%)	
*2017 European LeukemiaNet genetic risk stratification, n (%)*					
Favorable	6 (18.8%)	2 (20.0%)	3 (27.3%)	1 (9.1%)	.829
Intermediate	18 (56.3%)	6 (60.0%)	5 (45.5%)	7 (63.6%)	
Adverse	8 (25.0%)	2 (20.0%)	3 (27.3%)	3 (27.3%)	
*Remission status at the time of transplantation, n (%)*					
First complete remission (CR1)	15 (46.9%)	5 (50.0%)	4 (36.4%)	6 (54.5%)	.700
First partial remission (PR1), CR2	12 (37.5%)	3 (30.0%)	6 (54.5%)	3 (27.3%)	
> CR2, primary refractory AML	5 (15.6%)	2 (20.0%)	1 (9.1%)	2 (18.2%)	
*Hematopoietic stem cell transplantation comorbidity index (HCT-CI), n (%)*					
0	16 (50.0%)	6 (60.0%)	4 (36.4%)	6 (54.5%)	.712
1–2	11 (34.4%)	2 (20.0%)	5 (45.5%)	4 (36.4%)	
≥ 3	5 (15.6%)	2 (20.0%)	2 (18.2%)	1 (9.1%)	
*Karnofsky performance score, n (%)*					
< 80	4 (12.5%)	1 (10.0%)	1 (9.1%)	2 (18.2%)	.779
≥ 80	28 (87.5%)	9 (90.0%)	10 (90.9%)	9 (81.8%)	
*Donor type, n (%)*					
Matched sibling donor	10 (31.3%)	2 (20.0%)	3 (27.3%)	5 (45.5%)	.637
Matched unrelated donor	17 (53.1%)	7 (70.0%)	5 (45.5%)	5 (45.5%)	
Mismatched unrelated donor	4 (12.5%)	1 (10.0%)	2 (18.2%)	1 (9.1%)	
Haploidentical, mismatched related donor	1 (3.1%)	0 (-)	1 (9.1%)	0 (–)	
*Stem cell source, n (%)*					
Peripheral blood	30 (93.8%)	8 (80.0%)	11 (100%)	11 (100%)	.320
Bone marrow	1 (3.1%)	1 (10.0%)	0 (–)	0 (–)	
Cord blood	1 (3.1%)	1 (10.0%)	0 (–)	0 (–)	
*Conditioning regimen, n (%)*					
Myeloablative (MAC)	23 (71.9%)	8 (80.0%)	10 (90.9%)	5 (45.5%)	.047
Reduced intensity (RIC)	9 (28.1%)	2 (20.0%)	1 (9.1%)	6 (54.5%)	
*Chemotherapeutic regimen, n (%)*					
8 Gy TBI/CY2/Fludarabine	15 (46.9%)	7 (70.0%)	5 (45.5%)	3 (27.3%)	.090
FLAMSA-RIC/CY2/4 Gy TBI	9 (28.1%)	2 (20.0%)	1 (9.1%)	6 (54.5%)	
12 Gy TBI/CY2	5 (15.6%)	0 (–)	3 (27.3%)	2 (18.2%)	
8 Gy TBI/Fludarabine	3 (9.4%)	1 (10.0%)	2 (18.2%)	0 (–)	
*GvHD-prophylaxis, n (%)*					
Cyclosporine/MTX	26 (81.2%)	9 (90.0%)	10 (90.9%)	7 (63.6%)	.322
Cyclosporine/Mycophenolate mofetil	4 (12.5%)	1 (10.0%)	0 (–)	3 (27.3%)	
Post-transplantation Cyclophosphamide/tacrolimus/Mycophenolate mofetil	2 (6.3%)	0 (–)	1 (9.1%)	1 (9.1%)	
*Anti-Thymocyte Globulin (ATG), n (%)*					
Yes	23 (71.9%)	9 (90.0%)	9 (81.8%)	5 (45.5%)	.051
No	9 (28.1%)	1 (10.0%)	2 (18.2%)	6 (54.5%)	
*Donor-recipient cytomegalic-virus-status, n (%)*					
Negative/negative	16 (50.0%)	5 (50.0%)	8 (72.7%)	3 (27.3%)	.143
Negative/positive	6 (18.8%)	1 (10.0%)	0 (-)	5 (45.5%)	
Positive/positive	6 (18.8%)	2 (20.0%)	2 (18.2%)	2 (18.2%)	
Positive/negative	4 (12.5%)	2 (20.0%)	1 (9.1%)	1 (9.1%)	
*Female donor to male recipient, n (%)*					
Yes	5 (15.6%)	1 (10.0%)	2 (18.2%)	2 (18.2%)	.840
No	27 (84.4%)	9 (90.0%)	9 (81.8%)	9 (81.8%)	
*Grade II–IV acute GvHD, n (%)*					
Yes	12 (37.5%)	4 (40.0%)	4 (36.4%)	4 (36.4%)	.981
No	20 (62.5%)	6 (60.0%)	7 (63.6%)	7 (63.6%)	
*Acute GvHD grade (n* = *12)*					
Grade II	9 (75.0%)	3 (75.0%)	3 (75.0%)	3 (75.0%)	.558
Grade III	2 (16.7%)	1 (25.0%)	0 (–)	1 (25.0%)	
Grade IV	1 (8.3%)	0 (–)	1 (25.0%)	0 (–)	
*Survival in CR/relapse, n (%)*					
Survival in CR	29 (90.6%)	10 (100%)	9 (81.8%)	10 (90.9%)	.361
Relapse*	3 (9.4%)	0 (-)	2 (18.2%)¶	1 (9.1%) §	

Never cGvHD (chronic Graft-versus-Host Disease): never having cGvHD; Resolved cGvHD: all signs of clinically activity of cGvHD have disappeared, past history of cGvHD, no use of immunosuppression; Active cGvHD: physician reported inflammatory manifestations of cGvHD

*TBI 8 Gy/Cy2/Fludarabine:* 8 Gy TBI (four 2 Gy doses on two consecutive days), Cyclophosphamide 2 × 60 mg/kg on two consecutive days, Fludarabine 3 × 30 mg/m^2^ on three consecutive days

*FLAMSA-RIC/TBI 4 Gy/Cy2:* FLAMSA regimen (d -12 to d -9): Fludarabine 4 × 30 mg/m^2^, HD-Ara-C 4 × 2000 mg/m^2^, Amsacrine 4 × 100 mg/m^2^. Reduced intensity conditioning (RIC)-regimen after 3 days of rest: 4 Gy TBI on d-5 (two 2 Gy doses), Cyclophosphamide (2 × 40 mg/kg for MRD or 2 × 60 mg/kg for MUD, MMRD or MMUD) on d -4 to d -3 , Antithymocyte globulin 10 mg/kg for MRD or 20 mg/kg for MUD, MMRD, MMUD from d-4 to d-2, prophylactic donor lymphocyte infusions at day + 120 or 30 days after discontinuation of immunosuppression: 1–5 × 10^6^ CD3+ cells/kg

*TBI 12 Gy/Cy2:* 12 Gy TBI (Six 2 Gy doses on three consecutive days, d -7 to d -5), Cyclophosphamide 2 × 60 mg/kg on 2 consecutive days (d -4 to d -3)

*TBI 8 Gy/Fludarabine:* 8 Gy TBI (four 2 Gy doses on 2 consecutive days, d-5 and d-4), Fludarabine 4 × 30 mg/m^2^ (d -5 to d -2)

*At time of the evaluation: All patients with relapse in history were in complete remission of the initial diagnosed AML after donor lymphocyte infusions (n = 2 ¶) and therapy with azacitidine (n = 1 §) for a median time of 43.8 months (IQR 34.0, 98.6 months)

The participants (n = 32) and non-responders (n = 9) showed no differences in patient age at the time of allo-HSCT (p = .374), sex (p = 1.0), diagnosis (p = .401), ELN-risk classification (p = .434), remission status (p = .372), HCT-CI (p = 893), KPS (p = .559), GvHD prophylaxis (p = .402), donor type (p = .646), stem cell source (p = .544), the use of a female donor to a male recipient (p =. 568), donor recipient CMV status (p = .527), use of ATG (p = 1.0), the current cGvHD status (p = .248), length of follow-up (p = .712) and the status of relapse (p = 1.0). Participants and non-responders had similar aGvHD ≥ grade II in history (31.3% vs. 0.0%, p = .083).

### Chronic GvHD characteristics after transplantation

Table 2 shows the cGvHD characteristics and the NIH severity of cGvHD at maximum severity. Twenty-two patients (69%) developed cGvHD after allo-HSCT. The median time from allo-HSCT to the onset of cGvHD was 326 days (IQR 208, 601 days). At time of the evaluation, 11 patients (34.4%) had currently active cGvHD, 11 patients (34.4%) had resolved cGvHD and 10 patients (31.3%) never had signs of cGvHD.

**Table 2:** Chronic Graft-versus-Host disease characteristics

Chronic GvHD characteristics at time of onset of cGvHD (n = 22)	All	Resolved cGvHD (n = 11), n (%)	Active cGvHD (n = 11), n (%)	p-value
Median days from transplantation to onset of cGvHD (IQR)	326 (208, 601)	350 (224, 595)	240 (151, 723)	1.0
*Subcategories of cGvHD type at time of onset*				
Classic cGvHD	20 (90.9%)	10 (90.9%)	10 (90.9%)	1.0
Overlap cGvHD	2 (9.1%)	1 (9.1%)	1 (9.1%)	
*NIH severity of cGvHD at maximum severity*				
Mild	2 (9.1%)	1 (9.1%)	1 (9.1%)	.080
Moderate	11 (50.0%)	8 (72.7%)	3 (27.3%)	
Severe	9 (40.9%)	2 (18.2%)	7 (63.6%)	
*Organ manifestations of cGvHD at maximum severity*				
Cutaneous	16 (72.7%)	8 (72.7%)	8 (72.7%)	1.0
Oral	13 (59.1%)	5 (45.5%)	8 (72.7%)	.387
Eye	9 (40.9%)	4 (36.4%)	5 (45.5%)	1.0
Lung	5 (22.7%)	3 (27.3%)	2 (18.2%)	1.0
Gastrointestinal	4 (18.2%)	3 (27.3%)	1 (9.1%)	.586
Liver	4 (18.2%)	–	4 (36.4%)	.090
Vaginal	4 (18.2%)	2 (18.2%)	2 (18.2%)	1.0
Fascia	2 (9.1%)	–	2 (18.2%)	.476
Joint	2 (9.1%)	1 (9.1%)	1 (9.1%)	1.0
*Platelet count at cGvHD onset, thrombocytes/µl*				
Median (IQR)	224 (90, 325)	240 (90, 306)	175 (77, 387)	1.0
*Platelet count at cGvHD onset*				
< 100/nL	6 (27.3%)	3 (27.3%)	3 (27.3%)	1.0
≥ 100/nL	16 (72.7%)	8 (72.7%)	8 (72.7%)	

Data presented are n (%) unless otherwise indicated

cGvHD: chronic Graft-versus-Host disease; Resolved cGvHD: all signs of clinically activity of cGvHD have disappeared, past history of cGvHD, no immunosuppression; Active cGvHD: physician reported inflammatory manifestations of cGvHD; IQR: interquartile range; NIH: National Institutes of Health

### Socioeconomic characteristics

Table 3 shows the socioeconomic data at the time of the evaluation.

**Table 3:** Socioeconomic aspects

	All (n = 32), n (%)	Group 1: Never cGvHD (n = 10), n (%)	Group 2: Resolved cGvHD (n = 11), n (%)	Group 3: Active cGvHD (n = 11), n (%)	p-value
*Relationship*					
Single/never married	8 (25.0%)	1 (10.0%)	4 (36.4%)	3 (27.3%)	.246
Married/living with a partner	20 (62.5%)	6 (60.0%)	7 (63.6%)	7 (63.6%)	
Divorced/Widowed	4 (12.5%)	3 (30.0%)	0 (–)	1 (9.1%)	
*Employment*					
Working all-day	12 (37.5%)	4 (40.0%)	5 (45.5%)	3 (27.3%)	.209
Working part-time	5 (15.6%)	2 (20.0%)	3 (27.3%)	0 (-)	
Pensioners	15 (46.9%)	4 (40.0%)	3 (27.3%)	8 (72.7%)	
*School-leaving qualifications*					
Secondary modern school/intermediate school qualification	21 (65.6%)	6 (60.0%)	7 (63.6%)	8 (72.7%)	.816
Advanced technical college entrance qualification/A-levels	11 (34.4%)	4 (40.0%)	4 (36.4%)	3 (27.3%)	
*Care obligations to family*					
Yes	6 (18.8%)	3 (30.0%)	2 (18.2%)	1 (9.1%)	.471
No	26 (81.3%)	7 (70.0%)	9 (81.8%)	10 (90.9%)	

cGvHD: chronic Graft-versus-Host Disease; Never cGvHD: never having cGvHD; Resolved cGvHD: all signs of clinically activity of cGvHD have disappeared, past history of cGvHD, no immunosuppression; Active cGvHD: physician reported inflammatory manifestations of cGvHD

### FACT-BMT, FACT-Cog and PHQ-4 according to the chronic GvHD-status

Table 4 shows the mean scores of the FACT-Cog, FACT-BMT and PHQ-4. Using the classification of Van Dyk et al. [13], 4 patients (12.5%) had present cognitive impairments (PCI-score < 54) (Mean 52.3, SD 0.5). Patients of group 1 (never cGvHD) and group 2 (resolved cGvHD) showed similar values of FACT-CogPCI, FACT-CogQoL and FACT-CogPCA. Patients of group 3 (currently active cGvHD) had the lowest FACT-Cog values of all of the three groups.

Patients of group 1 (never cGvHD) and group 2 (resolved cGvHD) showed similar values of all FACT-BMT scores, while patients of group 3 (currently active cGvHD) had the lowest values of all of the three groups.

15.6% (n = 5) of patients were categorized as present of depression (PHQ2 ≥ 3) and 9.4% (n = 3) as present of anxiety (GAD ≥ 3). Regarding the psychological aspect, patients of group 1 (never cGvHD) and group 2 (resolved cGvHD) showed similar values of PHQ-4 scores, while patients of group 3 (currently active cGvHD) had the highest values.

**Table 4:** FACT-BMT, FACT-Cog and PHQ-4 according to cGvHD

Mean (SD)	All (n = 32)	Group 1: Never cGvHD (n = 10)	Group 2: Resolved cGvHD (n = 11)	Group 3: active cGvHD (n = 11)	F-value	p-value
*Functional Assessment of Cancer Therapy-Cognitive function (FACT-Cog)*						
Perceived cognitive impairment (PCI, score range 0–72)	63.9(6.3)	67.0(5.2)^a^	66.1(4.6)^a^	58.8(5.8)^b^	7.8	.002*
Comments from others (Oth, score range 0–16)	15.6(.84)	15.7(.9)	15.7(.5)	15.4(1.0)	.621	.545
Impact of perceived cognitive impairments on quality of life (QoL, score range 0–16)	14.2(1.7)	15.0(1.1)^a^	15.0(1.0)^a^	12.9(1.9)^b^	7.8	.002*
Perceived cognitive abilities (PCA, score range 0–28)	22.5(4.1)	24.8(3.2)^a^	23.8(3.0)^a^	19.2(3.9)^b^	8.4	.001**
*Functional Assessment of Cancer Therapy-Bone marrow transplantation (FACT-BMT)*						
Physical wellbeing (PWB, score range 0–28)	23.1 (4.8)	26.3 (2.1)^a^	25.8 (2.3)^a^	17.4 (3.2)^b^	39.9	< .001***
Emotional wellbeing (EWB, score range 0–24)	19.3 (3.8)	21.7 (2.5)^a^	21.0 (2.2)^a^	15.5 (3.1)^b^	17.0	< .001***
Social/family wellbeing (SWB, score range 0–28)	21.0 (3.8)	23.7 (1.0)^a^	22.5 (3.0)^a^	17.0 (2.6)^b^	23.6	< .001***
Functional wellbeing (FWB, score range 0–28)	21.1 (3.6)	22.8 (1.8)^a^	23.8 (1.3)^a^	16.8 (2.1)^b^	46.7	< .001***
Bone marrow transplant subscale (BMTS, score range 0–40)	30.8 (4.3)	33.4 (2.5)^a^	33.0 (2.7)^a^	26.4 (3.3)^b^	20.3	< .001***
FACT-G total score (score range 0–108)	84.6 (14.0)	94.4 (5.3)^a^	92.8 (6.7)^a^	67.5 (7.0)^b^	59.1	< .001***
FACT-BMT-Trial Outcome Index (TOI, score range 0–96)	75.1 (11.5)	82.4 (4.6)^a^	82.3 (5.0)^a^	61.4 (7.1)^b^	48.4	< .001***
FACT-BMT total score (score range 0–148)	115.5 (17.8)	127.8 (6.7)^a^	125.8 (7.9)^a^	93.9 (9.9)^b^	56.5	< .001***
Patient Health Questionnaire-4 (PHQ-4, score range 0–12)	1.94 (2.1)	1.3 (1.4)^a^	.91 (.94)^a^	3.6 (2.5)^b^	7.0	< .001***
PHQ-2 (score range 0–6)	1.09 (1.3)	.8 (.9)^a^	.45 (.82)^a^	2.0 (1.4)^b^	6.1	< .01**
GAD-2 (score range 0–6)	.84 (1.0)	.5 (.7)^a^	.45 (.52)^a^	1.5 (1.3) ^b^	5.0	< .05*

cGvHD: chronic graft-versus-host disease; Never cGvHD: never having cGvHD; Resolved cGvHD: patients with a past history of cGvHD, no immunosuppression; Active cGvHD: physician reported currently active inflammatory manifestations of cGvHD; FACT-BMT and FACT-Cog: higher scores indicate better quality of life; Patient Health Questionaire (PHQ-4): measurement of core symptoms of depression (PHQ-2) and anxiety (GAD-2)

Analysis of variance (ANOVA) with post-hoc Tukey-test: *p < .05, **p < .01, ***p < .001

Groups with different letters (a, b) differ significantly at 5% significance level by the Tukey-test. Equal letters do not differ by the Tukey-test

### Correlations between PHQ-4 and FACT-Cog and FACT-BMT

Table 5 shows the Spearman-Rho correlations between the PHQ-2 and the GAD-2 with the FACT-Cog and the FACT-BMT.

**Table 5:** Spearmen-Rho correlations between the PHQ-4, the FACT-Cog and the FACT-BMT

	PHQ-4	
	PHQ-2 depression	GAD-2 anxiety
*Functional Assessment of Cancer Therapy-Cognitive function (FACT-Cog)*		
Perceived cognitive impairment (PCI)	− .503**	− .490**
Comments from others (Oth)	− .286	− .438*
Impact of perceived cognitive impairments on quality of life (QoL)	− .550***	− .703***
Perceived cognitive abilities (PCA)	− .624***	− .487**
*Functional Assessment of Cancer Therapy-Bone marrow transplantation (FACT-BMT)*		
Physical wellbeing (PWB)	− .634***	− .389*
Emotional wellbeing (EWB)	− .555***	− .520**
Social/family wellbeing (SWB)	− .536**	− .183
Functional wellbeing (FWB)	− .639***	− .401*
Bone marrow transplant subscale (BMTS)	− .605***	− .374*

FACT-BMT and FACT-Cog: higher scores indicate better quality of life; Patient Health Questionnaire (PHQ-4): measurement of core symptoms of depression (PHQ-2) and anxiety (GAD-2)

*p < .05, **p < .01, ***p < .001

### Correlation of FACT-BMT and FACT-Cog

Table 6 shows the Spearman-Rho correlations between the FACT-BMT and the FACT-Cog scores.

**Table 6:** Spearmen-Rho correlations between the FACT-Cog and the FACT-BMT

	Functional Assessment of Cancer Therapy-Cognitive function (FACT-Cog)			
	Perceived cognitive impairment (PCI)	Comments from others (Oth)	Impact of perceived cognitive impairments on quality of life (QoL)	Perceived cognitive abilities (PCA)
*Functional Assessment of Cancer Therapy-Bone marrow transplantation (FACT-BMT)*				
Physical wellbeing (PWB)	.582***	.260	.594***	.559***
Emotional wellbeing (EWB)	.538**	.343	.707***	.536**
Social/family wellbeing (SWB)	.619***	.120	.311	.600***
Functional wellbeing (FWB)	.441*	.168	.549**	.432*
Bone marrow transplant subscale (BMTS)	.562***	.312	.510**	.528**

FACT-BMT and FACT-Cog: higher scores indicate better quality of life

*p < .05, **p < .01, ***p < .001

### FACT-BMT and FACT-Cog in comparison to normative data derived from the general population

**Fig. 1:**
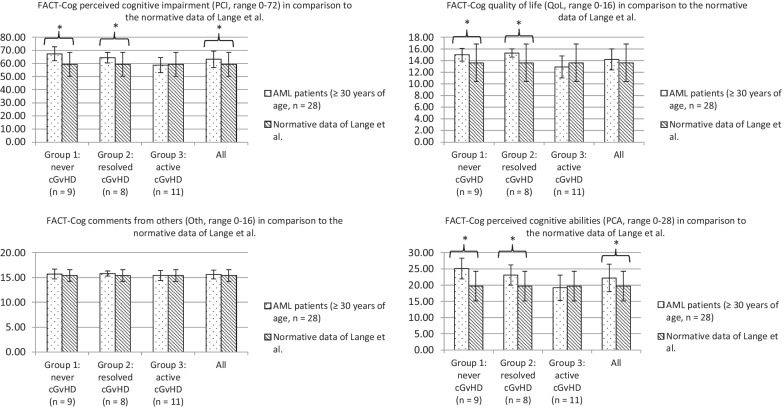
FACT-Cog according to cGvHD in comparison to the normative data of Lange et al. FACT-Cog: Functional Assessment of Cancer Therapy-Cognition; cGvHD: chronic Graft-versus-Host Disease; Never cGvHD: never having cGvHD; Resolved cGvHD: patients with a past history of cGvHD, no immunosuppression; Active cGvHD: physician reported currently active signs of inflammatory manifestations of cGvHD. Significance is indicated as p < .05 in brackets. Minimum clinically important differences (MCID) are indicated as *

Figure 1 illustrates the FACT-Cog scores of the AML patient sample (≥ 30 years of age, n = 28) and of all cGvHD groups (Group 1: never cGvHD; Group 2: resolved cGvHD; Group 3: active cGvHD) compared to the normative data of a French healthy population of Lange et al. [11]. The overall patient cohort (n = 28) had similar FACT-CogQoL, FACT-CogOth as well as higher FACT-CogPCI and FACT-CogPCA (accompanied by MCID) compared to the normative data of Lange et al. [11]. Patients of group 1 (never cGvHD) and group 2 (resolved cGvHD) had better FACT-Cog scores (PCI, QoL and PCA) compared to the normative data, accompanied by MCID. Patients of group 3 (active cGvHD) had similar FACT-Cog scores (PCI, QoL, Oth and PCA) compared to the normative data of Lange et al. [11].

**Fig. 2:**
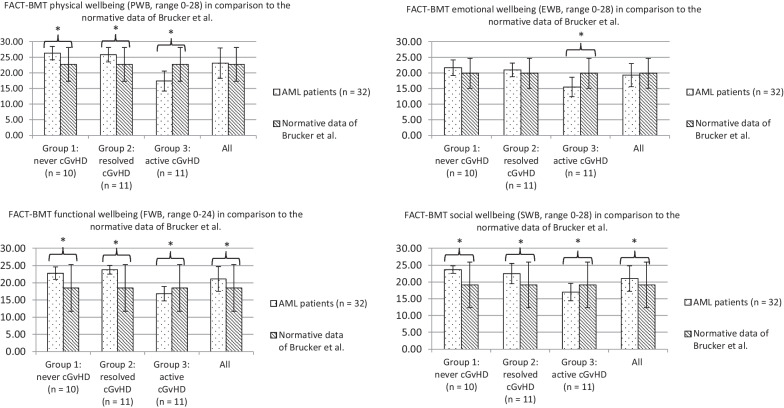
FACT-BMT/(FACT-G) according to cGvHD in comparison to the normative data of Brucker et al. FACT-BMT: Functional Assessment of Cancer Therapy-Bone marrow transplantation; FACT-G: Functional Assessment of Cancer Therapy-General; cGvHD: chronic Graft-versus-Host Disease; Never cGvHD: never having cGvHD; Resolved cGvHD: patients with a past history of cGvHD, no immunosuppression; Active cGvHD: physician reported currently active signs of inflammatory manifestations of cGvHD. Significance is indicated as p < .05 in brackets. Minimum clinically important differences (MCID) are indicated as *

Figure 2 depicts the FACT-BMT/FACT-G scores of the overall patient sample (n = 32) in comparison to the normative data of Brucker et al. (FACT-G) [10]. The overall population had similar (PWB, EWB) or better FACT-BMT scores (FWB, SWB) compared to the normative data. Patients with active cGvHD had significantly lower FACT-BMT scores (PWB, EWB, FWB, SWB) compared to the normative data of Brucker et al. [10], accompanied by MCID.

## Discussion

In this study, we analyzed the patient-reported CF and QoL of long-term transplant survivors after TBI-based-conditioning, which delivers a homogenous dose to the whole body and examined the impact of cGvHD on outcome parameters. Impairment of CF [3], commonly named chemo-brain, and reduced QoL [14] are common concerns following chemotherapy. Our findings indicate that cognitive impairment is no significant problem of long-term survivors after TBI-based-conditioning. The overall patient sample reported similar CF compared to the normative data of Lange et al. [11]. Even patients with active cGvHD had no worse FACT-Cog scores compared to the normative data which is in line with the fact that neurological manifestations of cGvHD are rare [15, 16]. We used normative data from a healthy French population [11] for comparisons with the FACT-Cog because we did not find reference values from a healthy German population. The normative data of Lange et al. [11] didn’t include patients < 30 years of age. We therefore excluded four AML patients (12.5%) < 30 years of age from the comparisons with the general population. The excluded patients had overall good FACT-Cog PCI (mean 68.8, SD 4.3), PCA (mean 24.8, SD 2.5), Oth (mean 15.7, SD 0.5) and QoL (mean 14.5, SD 1.3). Patients with active cGvHD reported a consistent pattern of deficits in physical wellbeing, emotional wellbeing, functional wellbeing, and social/family wellbeing. Similar associations of cGvHD and worse QoL, e.g. physical wellbeing, social wellbeing and mental wellbeing have been reported independently of the conditioning regimen [17, 18]. A previous study found similar self-reported QoL between patients who never had cGvHD and those whose cGvHD had resolved [14]. Our results confirm this observation. Noteworthy, the overall AML population had no worse FACT-BMT scores compared to the normative data of Brucker et al. [10].

In summary, our data indicate that no relevant impairments persist after resolution of cGvHD. Patients with resolved cGvHD and those who were never diagnosed with cGvHD had comparable long-term QoL and CF [14, 18, 19]. A reason for the overall good CF and QoL of the AML patients may be the relatively young patient population with a median age of 53 years (IQR 37, 62 years) at the time of evaluation. Moreover, the median time interval between allo-HSCT and the evaluation was 154 months (IQR 113, 191 months). Literature reports moderate impairment of QoL after allo-HSCT that returns to baseline levels as time from transplantation increases and more than 60% of patients have good to excellent QoL 1 to 4 years after allo-HSCT [20, 21]. Additionally, our results indicate that patients may have personal benefits after allo-HSCT for AML and report better patient-reported results because of a better appreciation for life [22, 23].

Our data show a negative association of depression and anxiety with QoL and CF. The relatively high frequency of anxiety and depression in long-term survivors with active cGvHD support the need for psychological support [14, 24]. We didn’t analyse fatigue or the impact of fatigue on QoL or CF. Long-term survivors of allo-HSCT can suffer from persisting fatigue several years after allo-HSCT and mental fatigue may be associated with cognitive dysfunction and reduced QoL [25, 26].

The low number of patients limits this study, which is the consequence of the long follow-up. In addition, we did not compare the outcome with patients, who received a conditioning regimen not containing TBI precluding an analysis of the specific impact of the latter. Nevertheless, we analysed a homogenous group of AML patients who were treated with TBI as part of the conditioning regimen in a consistent manner in a 20-years-period and did not observe a major impact of the transplant procedure itself on QoL except the impact of cGvHD which occurs independent of the conditioning regimen. Additionally, cGvHD, the major variable of interest was based on physician-reported institutional databases and National Institutes of Health Criteria. Of note, the overall response rate was high (78%) and we found no differences between participants and non-participants in baseline characteristics.

## Conclusion

The present study indicates that CF is not impaired after TBI-based conditioning, even in the presence of active cGvHD. However, active cGvHD has a significant impact on physical, emotional, functional and social wellbeing.

## Data Availability

The data used and analysed during the current study are available from the corresponding author on reasonable request.

## Abbreviations

TBI: Total body irradiation
allo-HSCT: Allogeneic hematopoietic stem cell transplantation
AML: Acute myeloid leukemia
cGvHD: Chronic graft-versus-host disease
CF: Cognitive function
QoL: Quality of life
FACT-BMT: Functional Assessment of Cancer Therapy-Bone marrow transplant
FACT-Cog: Functional Assessment of Cancer Therapy-Cognition Function
PHQ: Patient Health Questionnaire
MSD: Matched sibling donors
MUD: Matched unrelated donors
MMUD: Mismatched unrelated donors
MMRD: Mismatched related donors
KPS: Karnofsky performance score
HCT-CI: Hematopoietic cell transplantation-comorbidity index
ELN: European LeukemiaNet
ATG: Anti-thymocyte globulin
MV: Megavoltage
PWB: Physical wellbeing
SWB: Social wellbeing
EWB: Emotional wellbeing
FWB: Functional wellbeing
BMTS: Bone marrow transplant subscale
PCI: Perceived cognitive impairments
PCA: Perceived cognitive abilities
IQR: Interquartile range
ANOVA: Analysis of variance
MCID: Minimum clinically important differences
CR1: First complete remission
PR1: First partial remission
MAC: Myeloablative conditioning
RIC: Reduced intensity conditioning
